# The north-south policy divide in transnational healthcare: a comparative review of policy research on medical tourism in source and destination countries

**DOI:** 10.1186/s12992-020-00566-3

**Published:** 2020-04-22

**Authors:** Altaf Virani, Adam M. Wellstead, Michael Howlett

**Affiliations:** 1 Lee Kuan Yew School of Public Policy, National University of Singapore, 469C Bukit Timah Road, Singapore, 259772 Singapore; 2 Department of Social Sciences, Michigan Technological University, Houghton, USA; 3 Department of Political Science, Simon Fraser University, Burnaby, British Columbia Canada

**Keywords:** Medical tourism, Transnational healthcare, Policy research, Bibliometric analysis, Comparative review, North-south divide, Research-policy gap

## Abstract

Medical tourism occupies different spaces within national policy frameworks depending on which side of the transnational paradigm countries belong to, and how they seek to leverage it towards their developmental goals. This article draws attention to this policy divide in transnational healthcare through a comparative bibliometric review of policy research on medical tourism in select source (Canada, United States and United Kingdom) and destination countries (Mexico, India, Thailand, Malaysia and Singapore), using a systematic search of the Web of Science (WoS) database and review of grey literature. We assess cross-national differences in policy and policy research on medical tourism against contextual policy landscapes and challenges, and examine the convergence between research and policy. Our findings indicate major disparities in development agendas and national policy concerns, both between and among source and destination countries. Further, we find that research on medical tourism does not always address prevailing policy challenges, just as the policy discourse oftentimes neglects relevant policy research on the subject. Based on our review, we highlight the limited application of theoretical policy paradigms in current medical tourism research and make the case for a comparative policy research agenda for the field.

## Background

Health systems around the world are experiencing common challenges. Long wait times, tightening eligibility restrictions, narrowing service offerings, fiscal and human resource shortages, and changing demographic profiles and disease burdens have made it difficult for governments to effectively meet the healthcare needs of citizens. Medical tourism has emerged as one of many solutions, made possible by transnational mobility of information, ideas, expertise, people and capital, and growing consumerism in healthcare. Globalization has led to high levels of standardization of medical knowledge, practice protocols and technologies, allowing patients in high-income countries to obtain timely, high-quality and affordable treatment through healthcare providers in other countries. Developing countries too have tapped into the growing popularity of medical tourism to develop their economies. Healthcare providers in destination countries and their brokers have, for instance, taken advantage of the internet to market their services to international consumers [1]. Countries like India and Thailand have intentionally linked medical care with tourism in their messaging, to leverage their reputations as leisure destinations [2]. Healthcare providers in Argentina have pitched their own distinctive brand, through association with cultural motifs like the ‘tango’ in online advertisements [3]. The extent to which destination countries have benefited has hinged on their ability to integrate global technologies and knowhow with unique domestic advantages.

The use of medical tourism as a welfare and developmental strategy is nonetheless contested. Its role in bridging health system deficiencies, improving healthcare standards and stimulating local economies has been long recognized, but there is increasing awareness in recent years of the ways in which it can burden public resources and deepen health inequities, often at the cost of marginalized populations [4–7]. Medical tourism is therefore an important action arena for policymaking, necessitated not just by the need to encourage the industry, but also to minimize its socioeconomic discontents, and to address its ethical and legal challenges, both in countries from which medical tourists originate, and in those they seek healthcare [8–10].

Despite these concerns, most countries lack explicit strategies on deploying medical tourism towards defined policy goals. Decisions on the transfer of medical technologies, foreign investment in health, immigration laws for healthcare professionals and visa provisions for overseas patients are often negotiated as part of international trade agreements, with little coordination across policy subsystems, or convergence between sectoral policies despite their obvious interconnectedness [11, 12]. Consider the case of India, for example. While there have been concerted attempts to attract medical tourists, simplify visa processes, publicize domestic healthcare providers internationally and regulate medical travel agencies, these efforts have mostly focused on the promotional aspects and on streamlining the tourism experience, which while important, do not address macro policy issues such as capacity deficits, spillover effects and incongruities between sectoral policies [13, 14]. The background document for India’s National Health Policy makes no mention of medical tourism, nor does the ensuing policy engage with issues emerging from it, although India is a major regional hub, and the development of its private health sector has been greatly influenced by it [15, 16]. Likewise, the policy on M-visas is determined by the Ministry of External Affairs, with little input from the Ministry of Health and Family Welfare on absorption capacity and effects on local health systems.

Poor availability of data is another challenge [17]. A large number of medical tourists travel on ordinary tourist visas to avoid cumbersome bureaucratic procedures, keeping a significant portion of medical tourism activity outside the purview of policymakers who might otherwise find such information invaluable [18]. There are definitional variations and no standardized method for collating data on global medical travel [19]. National databases that serve to evaluate health policies and programs, do not typically capture information on the nature and quantum of transnational transactions in medical services. Even in Canada, which has a strong tradition of evidence-informed policymaking, the major databases maintained by the Canadian Institute for Health Information (CIHI) and Statistics Canada, do not provide information on publicly funded out-of-country care (OOCC) sought abroad privately as medical tourists [20]. The Canadian Longitudinal Study on Aging (CLSA) includes a variable on international travel for leisure, but not for healthcare – despite the fact that consumers of medical tourism services are typically elderly. As a result, policymakers often do not have the informational means and empirical foundations for mapping the contours of the industry, or for making reliable assessments of its systemic and network effects in source and destination countries, leading to patchy policymaking [21]. That this issue remains at the periphery of governmental strategic thinking and a neglected policy area despite its public significance, is surprising.

While recent research has identified global themes in medical tourism research [22, 23], transnational differences in policy and policy research on this subject have been less explored. Medical tourism occupies different spaces within the national policy landscapes of countries, depending on which side of the transnational paradigm they belong to, and how they seek to leverage it towards their goals. In this article, we draw attention to this policy divide through a comparative review of policy research on medical tourism in select source (Canada, United States [US] and United Kingdom [UK]) and destination countries (Mexico, India, Thailand, Malaysia and Singapore). These countries are among the major hubs of medical tourism activity and the focus of significant empirical research in the field in recent years. We identify themes that scholars have sought to address, and their relative prominence in policy-related research on these countries. Based on our review, we assess cross-national differences in policy and policy research on medical tourism against contextual policy landscapes and challenges, and identify research-policy gaps.

## Methods

**Fig. 1:**
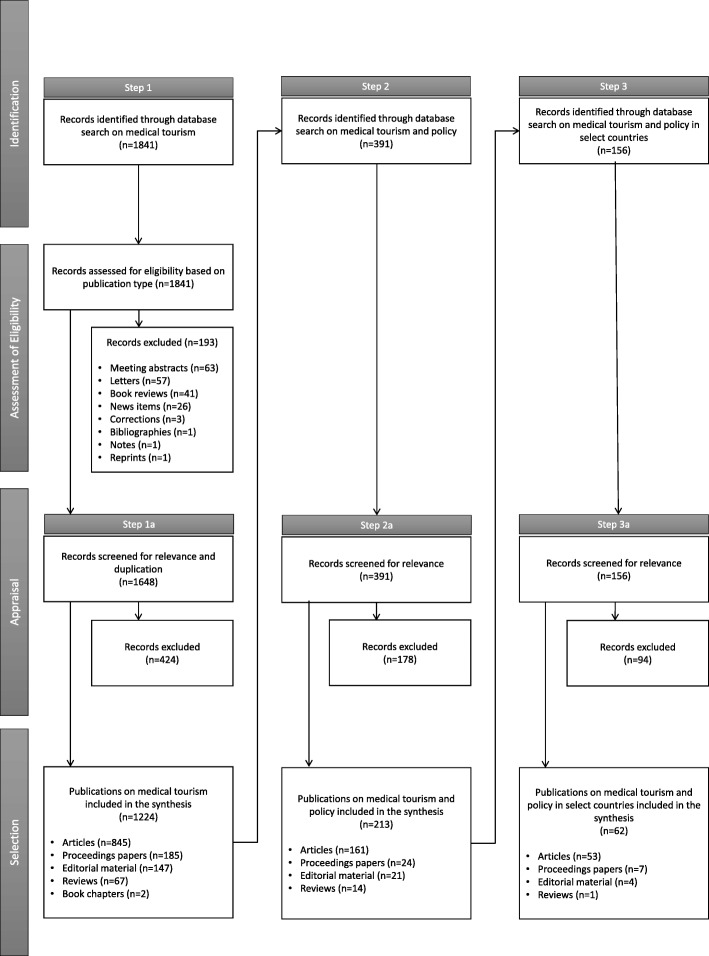
Steps in the review process

The review was conducted using the PRISMA (Preferred reporting Items for Systematic review and Meta-Analyses) process flow developed by Moher, Liberati et al. [24] (Fig. 1). A Boolean search of the Web of Science (WoS) Core Collection was performed between October and November 2018 to sequentially identify policy-related publications on medical tourism in the selected countries for all years till date. The search was limited to the WoS database due to the technical challenges of multi-source comparative analyses and to facilitate more in-depth analysis of the materials identified. We covered both open access and subscription based publications. We included journal articles, reviews, books, book chapters, conference proceedings, editorial material, trade publications and industry reports, but excluded reprints, book reviews, news articles, letters, meeting abstracts, short surveys, conference reviews, errata, bibliographies and notes. No language or geographical restrictions were applied to search results.

The initial search identified publications on medical tourism (step 1). The search was then repeated to isolate the subset of publications on policy (step 2). A final search was conducted to shortlist publications specifically referencing select source (Canada, US and UK) and destination countries (Mexico, India, Thailand, Malaysia and Singapore) (step 3).^1^ Records were screened for duplicity and relevance, and those found not relevant were removed following each search iteration. Relevance was assessed qualitatively through a review of publication titles and abstracts (or full texts when these were either ambiguous or insufficient) based on three criteria – whether the materials explicitly dealt with medical tourism, whether they (directly or indirectly) addressed policy-related aspects, and whether they were rooted in the contexts of the selected countries or contributed to their understanding. Publications that met all the three criteria were included in the final shortlist. Those only tangentially alluding to but not substantively engaging with them were excluded. The studies included in the bibliometric review based on appraisal of the context country, study objectives and main conclusions are outlined in Additional file 1 uploaded as electronic supplementary material. The bibliographic data was imported into a data visualizing software called *VOSviewer* to create and visualize a network map of research themes covered.

In addition, we searched Google and Google Scholar search engines, and database archives such as the Public Affairs Information Service (PAIS) Index, PolicyArchive, IssueLab and Factiva for grey literature on policy issues related to medical tourism in the selected countries (step 4). These included government agency reports, position papers, industry publications, thinktank analyses and news articles. Results from this last step (not shown in Fig. 1) were not included in the bibliometric review, but were rather used to supplement our interpretation of different problem and policy contexts, and juxtapose the identified policy research against them.

## Comparative country cases

**Fig. 2:**
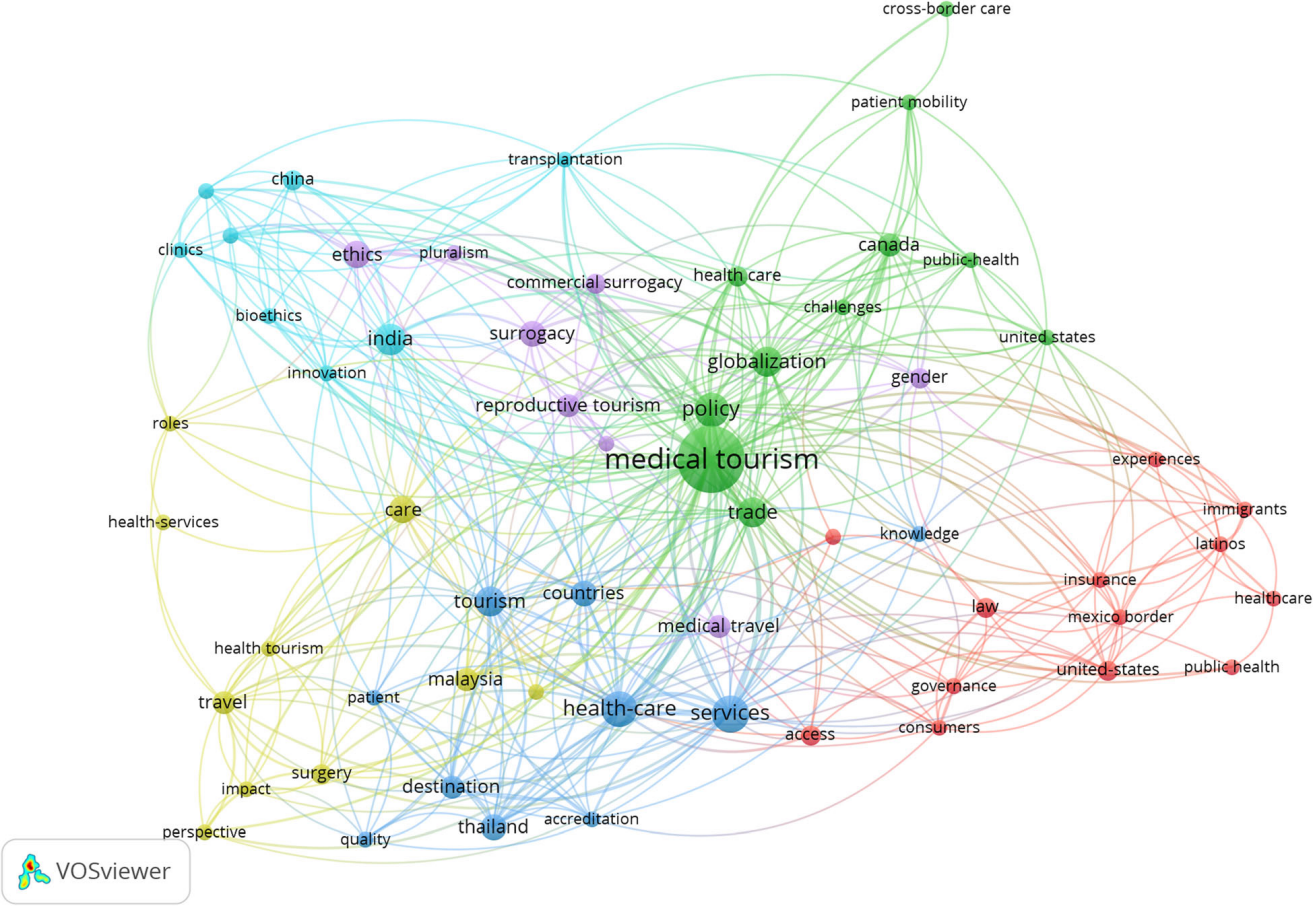
Co-occurrence network of keywords in policy-related publications on medical tourism in select source and destination countries. The figure shows the relatedness of author designated and auto indexed keywords (with at least three occurrences) based on how frequently they occur in the same publications. Nodes depict keywords and their linkages convey co-occurrence relationships. Larger nodes indicate keywords with more occurrences. Link strengths indicate the frequency with which they co-occur. Keywords that co-occur more frequently are clustered together in nodes of the same color

Out of the 1224 publications focused on medical tourism, 62 publications explicitly address policy with reference to one or more of the 8 selected countries (Canada, US, UK, Mexico, India, Thailand, Malaysia and Singapore). Figure 2 maps the key themes that scholars have sought to address, and their relative prominence in research with respect to each country. Table 1 juxtaposes this research against the key policy challenges that source and destination countries are faced with. Based on our review, we identify cross-national patterns in policy research, and highlight synergies and gaps in research and policy.

**Table 1:** Policy challenges of medical tourism in source and destination countries and issues addressed in policy research

Country	Key policy challenges	Issues addressed in policy research
Canada	Patient wait times Physician shortages Quality of care in private sector Effectiveness and safety of stem cell therapies Regulation of medical tourism companies Effects on provincial healthcare systems Access to private healthcare for residents Standards of care in destination countries	Institutional drivers of medical tourism Patterns of medical tourism and effects on preferential access Development of health and safety advisory for caregiver-companions Ethical and legal concerns of medical tourism Obligations of healthcare providers towards outbound patients Facilitation of medical tourism Physician concerns with care quality and continuity
US	High healthcare costs Cost-control challenges and moral hazard Disparities in access Selective coverage Effectiveness and safety of experimental therapies Regulation of medical and pharmaceutical industries Standards of care in destination countries	Institutional drivers of medical tourism Motivations for medical travel Transnational differences in policy goals Regulatory barriers Domestic medical tourism Legal protections for medical tourists Market for selective technologies and its ethical concerns Ethical and logistic challenges of transplant tourism Transparency in organ transplantation
UK	NHS cutbacks Shortage of healthcare professionals Immigration rules for healthcare professionals Patient wait times High cost of private healthcare Effects of treatment time guarantee Ethics of private sector contracting Access to medicinal cannabis Rights of UK citizens to healthcare in the EU Cross-border jurisdiction issues Healthcare coverage for migrants and visitors Revenue generation from medical tourism Effectiveness, safety and legality of experimental therapies Standards of care in destination countries Insurance protection for outbound medical tourists Spread of drug-resistant superbugs Effects of Brexit	Estimates of medical tourism flows Economic and health system implications Motivations for medical travel Rights and entitlements of migrants and visitors Access to abortion services and barriers to legal reform Regulation of international surrogacy, stem cell markets and organ trafficking Bilateral agreements in medical tourism
Mexico	Market for medical tourism and its economic potential Supply-side capacities and constraints Mexico’s role in global public health Medical risks and safety concerns	Use of Mexican medical providers by US patients, insurers and hospitals Cross-border health seeking by US Hispanic immigrants Supply-side drivers and impediments Effects of economic integration Binational insurance Effects of healthcare reforms on cross-border healthcare Regulation of stem cell treatment
India	Market for medical tourism and its economic potential Government’s role in promoting medical tourism Distortionary effects on health system and equity implications	Motivations for medical travel and patient experiences Medical tourism development Impact on health system and health workforce Ethical concerns and gaps in regulation of assisted reproduction, organ transplantation and stem cell treatment Bilateral agreements in medical tourism
Thailand	Market for medical tourism and its economic potential Competitiveness Government’s role in promoting medical tourism Distortionary effects on health system and equity implications	Perceptions of medical tourists and implications for destination choice Patient experiences Medical tourism development Economic and health system implications Regulation of assisted reproduction and its limitations
Malaysia	Market for medical tourism and its economic potential Competitiveness Government’s role in promoting medical tourism Distortionary effects on health system and equity implications	Perceptions of medical tourists and implications for destination choice Motivations for medical travel and care seeking behavior Medical tourism development Competitiveness Economic and health system implications Ethical and legal concerns Sustainable development Adaptation in organ donation legislation and transplant policy
Singapore	Patient satisfaction Competitiveness	Medical tourism development Competitiveness Economic and health system implications Adaptation in organ donation legislation and transplant policy

### Source countries

#### Canada

Universal coverage under the Canada Health Act (CHA) excludes certain services and classes of treatment, which have to be sought in the private market. Moreover, the health system is burdened with manpower shortages and long wait times for elective procedures. Consequently, over a million Canadians waited for treatment in 2018, forcing many to venture abroad at personal cost to access required healthcare [25, 26]. Concerns have been raised regarding the safety and quality of treatment in the private sector, both in Canada and in destination countries, and the regulation of intermediaries that facilitate such travel and treatment [27]. Comparatively, inbound flows are restricted due to limited private sector offerings, and opposition to Canada’s provincial hospitals catering to medical tourists due to implications for preferential access by Canadian residents under the publicly funded single-payer system [28].

The peculiar characteristics of the Canadian health system have been recognized as driving these distinctive patterns in transnational health seeking practices [29]. Yet, empirical research on their systemic drivers and effects is scarce. The objectives of most research are exploratory or descriptive, and focused on ethical and medico-legal issues arising from outbound medical tourism [30–34], contradictions between intent and policy [35, 36], and operational challenges [37–39]. This points to the relative nascency of policy research on Canadian medical tourism.

#### United States

The American health system suffers from major gaps in coverage, high costs of treatment, and disparities in access and health outcomes [40]. Many Americans travel overseas in pursuit of more affordable treatment, while wealthy clients from across the world flock to American hospitals to avail cutting-edge or experimental therapies. The private sector caters not only to foreign medical tourists, but also to domestic travellers [41]. It also facilitates international medical travel through accreditation and insurance services, travel intermediation and brokerage [21]. Medical tourism in the US has global commercial tributaries and revenue streams that entails exclusive networks and selective contracting with overseas low-cost providers, to foster competition on price and control costs [42, 43]. In areas like experimental therapies and biomedical innovation, cross-jurisdictional differences in regulatory regimes, and supply-side hegemonic networks of the medical industry, big pharma and the government, make it easier for suppliers to skirt stringent regulation and render demand-led consumer-driven governance approaches ineffective, unless accompanied by domestic advocacy, or policy initiatives in destination countries to trigger conformity by western suppliers [44–46].

Such analytical nuance and the range of practical solutions that have been devised can be attributed to the industry being better researched, and policy research being more evolved and contextually oriented to regional realities in the US, than for instance, in Canada. Researchers have studied how factors such as transnational regulations, international treaties and trade agreements [47, 48], domestic healthcare reforms [49], structural disparities [29, 50, 51], market forces [29, 50], personal preferences of medical tourists [52, 53] and destination country characteristics [53, 54] affect cross-border healthcare. It has been argued that the focus on cost savings in policy debates in the US neglects such concerns as access, equity, quality and capacity, which occupy the centre stage in policy thinking in countries like Canada and the European Union [29, 55]. Researchers have, for instance, highlighted how regulatory approaches in the US tend to disregard, and sometimes aggravate concerns about patient protection, patient choice, and ethically questionable technologies [56, 57], but selectively adopt a moral high ground or leave regulatory ambiguity when convenient [58, 59].

#### United Kingdom

Some of UK’s issues are similar to Canada’s. Cutbacks in National Health Service (NHS) funding, shortage of healthcare professionals, long wait times, treatment time guarantees, drug controls and the high cost of private healthcare, drive thousands of patients to healthcare destinations in Eastern Europe and Asia every year. Hanefeld and Horsfall [60] and Lunt, Smith et al. [61] have examined some of these motivations for travel abroad. Private sector contracting has sought to relieve pressure on the public healthcare system, but raised ethical and equity concerns. The UK has had to additionally contend with regulatory differences among its constituent countries, and challenges arising from EU membership, such as the rights of UK citizens in the EU, questions of extraterritorial application of domestic laws and trans-regulatory disparities. For example, researchers have drawn attention to legal barriers in accessing abortion services in Northern Ireland [62], weaknesses in the regulation of international surrogacy [63], regulatory deficiencies which allow circumvention of statutory restrictions on organ trafficking [64], lack of conceptual harmonization in the regulation of stem cell innovation [45] and implementation overreach in NHS practices towards EU visitors [65].

In addition, UK’s experience with medical tourism has drawn attention to common concerns about the effectiveness, safety and legality of experimental therapies, and standards of care in destination countries. Internally, it has highlighted gaps in insurance protection for outbound medical tourists, public health challenges arising from cross-border spread of antibiotic-resistant *superbugs*, and the inadequacy of data on international medical travel. There have been calls for moving towards regional or bilateral frameworks to redress such issues, but political considerations and electoral expediency have posed challenges [66].

Public debates have been shaped by political rhetoric largely centred on immigration, access to free healthcare for migrants and visitors, and the role of the NHS. While there is little evidence that this compromised the care of vulnerable populations, it has created confusion and uncertainty among care providers and consumers [67]. Many assumptions driving policy on these issues have been contested by recent research. For example, data on cross-border flows shows that medical tourism accounts for only a fraction of the NHS health spending, and that concerns about it burdening taxpayers are misplaced and overstated. To the contrary, it is a significant contributor of revenue. Moreover, the UK is a net exporter of medical tourism, and by implication a social beneficiary of cross-border healthcare [68]. In the wake of the uncertainty surrounding Brexit, there are questions about how medical tourism and future coordination with EU members on healthcare might be affected. There has been conjecture about possible scenarios, but no externally facing analysis or investigation of its likely effects and implications is available.

### Destination countries

#### Mexico

Mexico is the world’s second largest medical tourism market, after Thailand, and annually attracts over a million medical tourists, particularly from the US and Canada. The focus of both federal and regional governments has been on policies to promote Mexican provinces as destinations for low-cost medical tourism, and developing medical, hospitality and recreational infrastructure to cater to inbound flows. These include training of healthcare professionals with bilingual language proficiency, increasing the number of accredited private hospitals, collaborating with overseas insurers and healthcare providers to establish referral and feeder networks, and creating medical clusters to tap unused capacities in existing private hospitals to service foreign clients.

While these initiatives have helped, structural barriers such as high market entry costs, visa restrictions and poor portability of health insurance have impeded sector development [69]. US Hispanic immigrants constitute a major segment of medical tourists in Mexico, partly due to care perceptions and personal preferences, but also due to the difficulties in obtaining required healthcare in the US [51, 70]. Researchers have suggested how cross-border insurance plans, immigration and healthcare reforms, and regional economic integration and trade agreements can remove some of these barriers, expand healthcare coverage and address regulatory grey zones [48–50].

#### India

India ranks fifth on the global Medical Tourism Index, second in Asia. Its private sector has made huge investments in hospital infrastructure, high-end medical technologies, network logistics and supply chains, to make state-of-the-art healthcare available to foreign clients at low cost [71]. The government has aided development through policies incentivising the private sector and clearing regulatory hurdles. This includes promotional campaigns, simplification of visa rules, financial support to medical tourism providers, registry for accredited agents to discourage touting, and a National Medical and Wellness Tourism Board (NMWTB) to formulate policy and guidelines for the sector. Debata, Sree et al. [72] have identified the key enablers of medical tourism in India and their effects.

However, there has been little interest among policymakers in regulating private healthcare providers to manage quality and safety concerns, or addressing staff shortages and resource pressures in the public sector due to the insidious transfer of human resources and state subsidies to the private sector through both diffusionary and policy-led mechanisms [21, 73–77]. In areas where attempts to regulate have occurred, as in the case of assisted reproductive technologies, organ transplantation and stem cell research, regulatory instruments have been found to be inadequate, ambiguous, and oftentimes contradictory [46, 78–83]. Such weaknesses in regulatory frameworks and domestic political constraints have hampered regional and bilateral cooperation in the sector [66].

#### Thailand

Thailand is the world’s top medical tourism destination and a major regional hub for patients from East and South-East Asia, competing with Japan, South Korea and Malaysia for regional dominance. High clinical standards and service quality at low cost, combined with extensive tourism infrastructure and leisure options, have created a strong preference for Thailand among medical travellers [84–86]. Medical tourism is part of the core business model of Thai private hospitals. Moreover, Thailand is regionally unique, in that it has a comprehensive health system architecture with high government investment in medical education and public health, a tightly regulated healthcare environment and universal health coverage [87]. Medical tourism is viewed by policymakers as a means to leverage the strengths of the health system to offset the budget burden towards social spending. The Royal Thai Government has aided this sector by placing it high on the policy agenda, easing visa restrictions for medical tourists, granting long-stay visas for travellers from ASEAN Plus Three countries (China, Japan and South Korea) and via promotional campaigns. Bochaton [88] has examined these developments and highlighted some of the key socio-political and economic drivers that have contributed.

Nonetheless, there are concerns about the effects of medical tourism on the health system, such as through internal migration of healthcare professionals, burdens created by foreign retirees seeking long-term end-of-life care, and cost inflation for the local population [21, 87, 89–91]. A range of policy prescriptions have been offered – integrated human resource planning, increased private sector contribution to human resource development, lifting restrictions on the recruitment of international medical graduates, levying a medical tourist tax to transfer some of the commercial gains from medical tourism to the health system, and reviewing policies for long-stay tourism. The government has taken some countermeasures, such as fee schedules for controlling medical costs, but many of the structural issues remain unaddressed. Besides, while bona fide regulatory efforts in areas such as commercial surrogacy and assisted reproduction have succeeded in clarifying legal positions and pre-empting litigation, they have presented new ethical and practical challenges, such as restrictions on same sex couples seeking surrogacy arrangements, and the use of human embryos for research [92].

#### Malaysia

Malaysia offers a clear pricing advantage as compared to neighbouring Singapore. However, there have been apprehensions that it may not enjoy a distinctive brand recognition like its regional competitors, as it has to compete with countries like India and the Philippines on cost, and with others like Thailand and Singapore on quality and consistency [93]. The Malaysian government has implemented a slew of measures, from *Green Lane* clearance to make it easier for medical travellers to seek healthcare in Malaysian hospitals, to industry recognition awards, accreditation programmes, infrastructure development and global promotion of brand Malaysia.

All the same, policymakers have failed to consider the socio-economic diversity, social networks and contextual characteristics of clients from regional markets like Indonesia and international retiree migrants in Malaysia in their zeal to appeal to the global medical tourist [94, 95]. Ormond and Sulianti [94] have attributed this disparity to *Northerner bias.* Others like Manaf, Hussin et al. [96] have suggested this may be a deliberate strategy by policymakers to avoid becoming overdependent on single source markets. Moreover, the development of Malaysia’s medical tourism industry has been concomitant with the rise of the private sector in health, and the withdrawal of the state from its welfarist agenda [97, 98]. Policies for medical tourism have tended to focus on maximizing indirect economic gains, while more obvious health system implications have been neglected [87, 99]. Failure to devise policies to address such concerns has resulted in critical regulatory gaps, producing less desirable and often inequitable outcomes [100–102]. The Malaysian case highlights the need for more contextually situated, and socially aligned policy and practice that is sensitive to, and has the support of diverse domestic stakeholders [103, 104].

#### Singapore

Medical treatment in Singapore costs significantly more as compared to other destination countries. Minimal government oversight of tourism activity and lack of stringent price controls in the private sector have contributed to cost inflation. This combined with other incidentals such as the high cost of living, makes it difficult for Singapore to maintain its competitiveness against regional rivals like Malaysia, India and Thailand, given how aggressively these countries have worked to develop their infrastructure and capacities, while operating at much lower costs. As a result, Singapore’s attractiveness as a medical tourism destination has somewhat diminished, because overseas patients have been diverted to more competitive markets in the immediate neighbourhood.

While the government provides only minimal direct assistance to medical tourism providers unlike in some other destination countries, it has supported the sector through targeted economic and industrial policy and incentive regime, broad-based supply-side reforms and a strong regulatory framework for corporate governance and legal regulation of firms. This has helped create a business-friendly climate, provided the required institutional scaffolding to ensure sound management accountability and sustainable development of the sector, and facilitated public-private collaboration and strategic cooperation between commercial players to establish a range of critical competencies. These include: 1) biomedical research and innovation labs such as the Agency for Science, Technology and Research (A*STAR) and SingHealth’s Investigational Medicine Unit; 2) the interagency government-industry partnership known as *Singapore Medicine* that coordinates national policy and strategy on medical tourism; 3) a globally-oriented medical education system with residency programs structured on American medical licensing norms, scholarships for physicians to train overseas and global partnerships such as the Duke–NUS Graduate Medical School; 4) healthcare accreditation systems based on international standards such as the Joint Commission International (JCI) and those mandated by local accrediting agencies like the Singapore Accreditation Council (SAC), the Ministry of Health and the Health Promotion Board (HPB); and 5) support sectors such as Meetings, Incentives, Conventions, and Exhibitions (MICE), information technology, and smart infrastructure [87, 93, 105, 106]. Thus, despite its cost disadvantage, Singapore has managed to create a unique niche for itself through purposive, policy-driven interventions that have built on and contributed to the development of technological superiority, technical efficiency, service quality and a comprehensive services ecosystem, which have helped better address concerns on safety and quality of care, and deliver superior patient experience relative to regional competitors.^2^

Though public hospitals are under government ownership, they are managed autonomously, and compete for patients in the open market. This induces competition on quality, but also provides avenues for cross-subsidization and economies of scale [87, 98]. Unlike Malaysia, where the public and private sectors have grown further apart, in Singapore, they have converged to offer relatively similar quality and scope of services, making public hospitals equally attractive to medical tourists. Programs and policies for medical tourism are therefore as likely to influence the behaviour of public hospitals, as of those in the private sector. While this can potentially translate to efficiency gains for public hospitals, theoretically it can also produce adverse public outcomes and compromised care for local residents, unless calibrated through policy. To minimize such distortions, the government has implemented stringent administrative controls to regulate the quality of care in public hospitals and adopted creative human resource policies. For instance, Singapore has successfully managed to attract high quality health workers from neighbouring countries like India, Pakistan, Sri Lanka, Malaysia, Myanmar and the Philippines over the years through international recruitment efforts, offer of competitive public sector salaries, and training and career development opportunities [87]. More recently, the government has attempted to reverse its previous policy position on public sector participation in medical tourism and restricted public hospitals from actively marketing themselves via foreign agents to address critical shortages in hospital beds and prioritize local healthcare needs such as those of an aging population, over those of foreign patients. While this may have realigned the internal medical tourism market in favour of private sector operators such as Parkway Holdings and the Raffles medical group which depend on medical tourists to feed their revenue streams and expand their domestic footprint, the public mandate of public hospitals has come to be better recognized in policy discourse, and better protected through government action.

A similarly pragmatic approach can be seen in Singapore’s organ donation legislation and transplant policy, where the government has attempted to draw a balance between promoting self-sufficiency in organ supply on the one hand, and minimizing the risk of exploitation on the other. Amendments to the Human Organ Transplant Act (HOTA) have relaxed donor eligibility norms to allow paired matching for the exchange of organs to increase the chances of better transplant outcomes. They also permit compensation to living donors for direct and indirect costs associated with organ donation through their medical savings accounts to minimize the risk of organ trade. The policy exhibits features of adaptive policy change geared towards addressing both the practical and ethical challenges of transplant tourism [102, 107]. Such pragmatism, while not unique to Singapore, differentiates it from other destination countries in its league, in terms of the responsiveness, responsibility and purposive intent of policymaking.

### Study limitations

Our assessment of the country cases is constrained by methodological weaknesses inherent to the design of bibliometric studies and qualitative comparative analyses of this kind. Some of these issues are discussed below to help the reader better contextualize the results and our conclusions.

Our search was limited to the WoS database, and did not include discipline-specific databases such as EconLit (economics), PubMed (biomedical and life sciences) and PsycINFO (psychology and behavioural sciences). Nonetheless, a significant proportion of their publications are cross-indexed in WoS, and therefore, while our search cannot be claimed as all-inclusive, it provides us with a reasonably representative snapshot of the state of medical tourism and policy research in the selected countries. Moreover, WoS has a significant overlap with Scopus, which is a larger database. Both tend to underrepresent the social sciences, arts and humanities, and both favour publications in the English language, which are key limitations [108].

The study uses Boolean search to identify publications on medical tourism, filter out those that do not directly refer to policy aspects and further shortlist those dealing with policy specifically in the context of the selected countries. While we employed keyword combinations and search iterations including synonyms and truncation or wildcard searches to get more comprehensive results, it is but inevitable that some relevant literature may have fallen through the cracks, while less significant works may have been included. For instance, we used a fairly narrow and explicit operationalization of policy research as works referring to *policy*, *governance*, *regulation* or *reform*. This might have caused some of the less explicit yet policy-relevant works to be overlooked in our search.

Further, given the subjective nature of the appraisals (steps 1a–3a), the pooled shortlist represents the authors’ collective judgement about the relevance of included works. Such subjectivity is likewise reflected in the nature of comparative lesson-drawing and gap-assessment presented in this paper. While some subjectivity is inevitable, we acknowledge that it affects replicability. Our aim was to balance the objectivity of a keyword-based search strategy to enhance replicability, with the application of mind for the determination of relevance, to improve the quality of the shortlist. Moreover, the objectives of our study are exploratory, not explanatory. The paper does not seek to establish causal connections, but rather to expose gaps in and between research and policy. A partly subjective approach was necessary for meaningful appraisal of the literature from this perspective.

Lastly, the distinction between source and destination countries in this paper is purely instrumental, as many countries are concurrently both providers and consumers of medical tourism for different services and to different degrees. For example, patients from India routinely travel to the US to benefit from newer medical technologies such as in stem cell and cancer research, while American patients visit India to overcome coverage issues and avoid high costs of treatment in the US. Such overlaps make the task of identifying country-specific policy challenges more complex and render source-destination policy comparisons muddled.

## Conclusions

These limitations notwithstanding, some interesting comparative insights are offered by the study. Firstly, we see major differences in national policy concerns, not only between source and destination countries, but also among them, given their unique socio-political contexts, developmental pressures and policy challenges. The diversity in policy research reflects their different realities (Table 1). How policymakers approach issues of cross-border healthcare, depends on which side of the transnational divide countries are located on, and how they seek to leverage the potential of medical tourism for the achievement of national policy goals.

Policymaking in destination countries is mostly directed towards developing supply-side capacities and competitiveness, without being particularly attentive to the distortionary effects on local health systems and the regulatory weaknesses that engender them. For instance, the governments of Mexico, India, Thailand and Malaysia have focused much of their policy attention, economic resources and governance capacities on promoting their provinces as destinations for low-cost medical tourism, incentivising private sector operators to strengthen medical and tourism infrastructure for medical tourists, and addressing regulatory hurdles for investors and consumers, often neglecting the health needs and wellbeing of their own populations, and the safety of medical tourists.

Source country policies, on the other hand, are designed to facilitate outbound medical travel to overcome domestic health system deficiencies and assure care standards in destination countries, while doing little to address the discriminatory application of ethical and regulatory norms to medical travel to those countries, or fix the issues that drive the exodus. Policymaking in the US, for example, has focused less on resolving the structural issues that limit healthcare access and coverage within the US, and more on establishing cross-border insurance mechanisms, transnational cost-saving arrangements and global networks of accredited healthcare providers to enable travel for treatment overseas. In the UK, a recent amendment to Northern Ireland’s abortion law has now made abortion services universally accessible. At the same time, however, both Canadian and UK laws for the regulation of transnational surrogacy and transplant tourism remain weak and ineffective in protecting surrogate mothers and organ donors in destination countries from exploitation.

The lop-sidedness in government priorities has skewed the narrative on medical tourism from that of a catalyst for socioeconomic development, health sector improvement and universal health coverage to a vehicle for economic growth and healthcare outsourcing. This divergence has created policy overlaps, tensions and fault lines between the *Global North* and *South*. While there is cross-national directional convergence in efforts to facilitate the travel of medical tourists, there remain critical gaps that cause treatment complications and hamper continuity of care in source countries, disadvantage vulnerable groups in destination countries, and create health inequities between source and destination countries. Addressing these requires the development of a global framework for the collaborative governance and enforcement of transnational processes of medical tourism, and mechanisms for policy coordination and regulatory convergence between source and destination countries to ensure policy alignment. It follows therefrom that policymakers need to explicitly consider what they seek to achieve through medical tourism in the context of their developmental goals, and purposively design a policy architecture that optimizes their gains and minimizes risks across the value chain. In source countries, this might, for example, involve legislative changes to facilitate extraterritorial application of protective provisions to prevent exploitation, and rationalization of regulatory restrictions and health system reforms to reduce the need for citizens to travel abroad for treatment. In destination countries, it might include cross-subsidization arrangements to transmit resources internally, incentive and regulatory regimes to control brain drain, and capacities to assure care quality and continuity through standardized treatment protocols, certification systems and networked electronic medical record exchange. It is notable that Singapore has been defter at navigating this divide. It has better addressed source country concerns on safety and quality of care as compared to its peers, and at the same time taken steps to insulate its health system from the unintended consequences of medical tourism. Its inward-focused and pragmatic policy approach may have potentially contributed to shielding it from some of the same problems that other destination countries have encountered, even as it has successfully managed to maintain its unique market position within the region.

Second, we find that research and policy are not always congruent. There are several areas where there are gaps from research being out of step with prevailing policy concerns. For instance, policy research in Canada is focused on drawing attention to the ethical and medicolegal concerns of medical tourism, and its undermining effects on access and equity, but does not adequately address the inherent weaknesses of the Canadian healthcare system that compel Canadians to seek treatment overseas, or the regulatory underpinnings that give rise to ethical conflicts. Likewise, there are areas where the policy discourse has failed to take heed of relevant policy research. In the UK, resistance to skilled worker immigration despite the high dependence of the NHS on foreign medical professionals, and the narrative driving policy against healthcare coverage for migrants despite lack of empirical evidence on systematic abuse, is a case in point. While it is well-recognized that policy and research seldom fully converge since the political economies that shape them operate autonomously and are slightly different, major incongruencies, like those mentioned above, can be mediated through better channels for collaboration and communication between researchers, policymakers and interest groups. Internationally, and especially in the health sector, knowledge platforms and policy labs are playing an increasingly important role in widening the evidence base for public policy through analytical inputs to help governments formulate better policy responses, building trust and confidence among diverse stakeholders, and co-producing research that is relevant to policymaking needs [109, 110]. Such initiatives in the medical tourism sector could go a long way in strengthening research-policy linkages, addressing obvious gaps in research and policy, and facilitating effective uptake of research by governments. Canada, for example, has a strong tradition of evidence-informed policymaking, and initiatives such as the McMaster Health Forum have contributed immensely to deliberative dialogues between health researchers, providers, managers and policymakers to optimize the use of research evidence in policymaking and practice [111]. These mechanisms can be leveraged for greater coordination between medical tourism policy and research. Beyond research-policy linkages, UK’s example of how political considerations can sometimes get the better of empirically-grounded policy advice, highlights the need for greater technocratic control over decision-making to the extent possible in democratic systems. While the role of political triggers in policymaking cannot be ignored, formal institutionalization of research into policymaking processes can help better insulate them from extraneous political influences and minimize political risk.

**Fig. 3:**
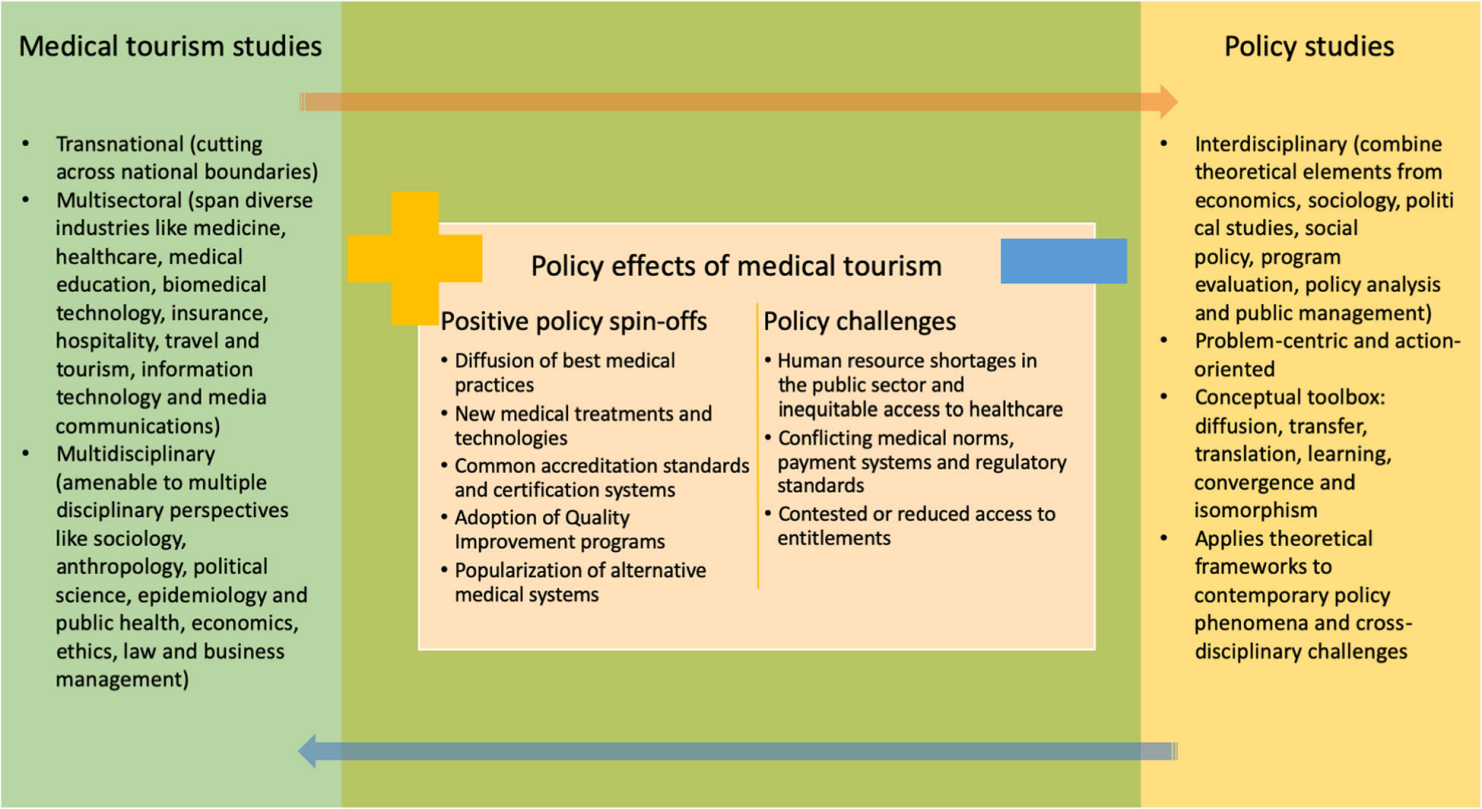
The intersection of medical tourism and policy studies

Our final point relates to the amenability of medical tourism research to a comparative policy perspective (Fig. 3). Consider, for example, the processes of diffusion that have helped countries supplying medical tourism services to optimize costs, upgrade technology, develop skills and knowhow, strengthen capacity and raise quality standards. The contagious character of globalization is likewise responsible for the widespread acceptance of the western biomedical concept of health, replication of clinical best practices, research advancements in new treatments and technologies, establishment of accreditation standards and certification systems, adoption of quality improvement methodologies by healthcare organizations, and cross-pollination of alternative systems like Homoeopathy in the Asian subcontinent, and Ayurveda and Yoga in the western world. Medical tourism has accelerated such diffusionary phenomena, which have in turn fueled its growth. In other cases however, it has slowed or reversed existing trends such as brain-drain, as working conditions back home have become more attractive. Such reversals have shifted the loci of existing policy problems and transplanted them to other pockets of the subsystem, requiring a different set of policy responses. For example, healthcare workers in countries like India, Malaysia and Thailand are increasingly migrating to the private sector to service foreign clients, leading to human resource shortages in the public health system, and forcing a rethink of domestic regulatory policies and human resource practices. Medical tourism has also brought to the forefront a number of practical difficulties, ranging from incompatibilities in medical norms and payment systems, to issues of contested citizenship and non-transferability of entitlements [8, 112–115]. Such fault lines typically arise from cross-border heterogeneity in policy regimes, poor regulatory coordination and lack of cooperative frameworks. Yet, there is limited application of theoretical policy paradigms and comparative analysis in current medical tourism research. This article makes a plea for a comparative policy research agenda for medical tourism that recognizes the need for holistic evaluation of its differential effects in source and destination countries, acknowledges the diversity in national policy concerns and priorities, and deploys appropriate policy frameworks and theories to help discern its various challenges.

## Supplementary information

**Additional file 1.** Publications included in the bibliometric review based on appraisal of context country, study objectives and main conclusions.

## Data Availability

The datasets used and/or analysed for the current study are available from the corresponding author on reasonable request.

## Abbreviations

ASEAN: Association of South East Asian Nations
A*STAR: Agency for Science, Technology and Research
CHA: Canada Health Act
CIHI: Canadian Institute for Health
CLSA: Canadian Longitudinal Study on Aging
EU: European Union
HOTA: Human Organ Transplant Act
HPB: Health Promotion Board
JCI: Joint Commission International
MICE: Meetings, Incentives, Conventions, and Exhibitions
MM2H: Malaysia My Second Home
NHS: National Health Service
NMWTB: National Medical and Wellness Tourism Board
OOOC: Out-of-country care
PAIS: Public Affairs Information Service
PRISMA: Preferred reporting Items for Systematic review and Meta-Analyses
SAC: Singapore Accreditation Council
UK: United Kingdom
US: United States
WoS: Web of Science

## Footnotes

1 We employed the following search string in step 1 to identify publications referring to medical tourism in their titles, abstracts or keywords: “medical tour*” OR “health tour*” OR “healthcare tour*” OR “surgical tour*” OR “transplant tour*” OR “cosmetic tour*” OR “reproductive tour*” OR “abortion tour*” OR “wellness tour*” OR “medical travel” OR “health travel” OR “healthcare travel” OR “surgical travel” OR “cosmetic travel” OR “reproductive travel” OR “wellness travel”. Policy-related materials were demarcated in step 2 with the following search: (“medical tour*” OR “health tour*” OR “healthcare tour*” OR “surgical tour*” OR “transplant tour*” OR “cosmetic tour*” OR “reproductive tour*” OR “abortion tour*” OR “wellness tour*” OR “medical travel” OR “health travel” OR “healthcare travel” OR “surgical travel” OR “cosmetic travel” OR “reproductive travel” OR “wellness travel”) AND (“policy” OR “policies” OR “regulat*” OR “governance” OR “reform”). The syntax from step 2 was used in conjunction with the following search terms: “Canad*” OR “U.K.” OR “United Kingdom” OR “Brit*” OR “England” OR “U.S.” OR “United States” OR “America” OR “India” OR “Thai*” OR “Malay*” OR “Singapore” OR “Mexic*” to identify country-specific policy research in step 3.

2 Singapore was ranked top among seven Asian medical destinations in terms of patient experience but least attractive on cost considerations as per an index developed by AB Bernstein and Global Health and Travel in 2017.
