# Supplementary material for: The north-south policy divide in transnational healthcare: a comparative review of policy research on medical tourism in source and destination countries
Source: Global Health. 2020 Apr 22;16:37. doi: 10.1186/s12992-020-00566-3 (PMC7178960; doi:10.1186/s12992-020-00566-3)
Supplement: Supplementary file 1 — Additional file 1. Publications included in the bibliometric review based on appraisal of context country, study objectives and main conclusions. [file 12992_2020_566_MOESM1_ESM.docx]

Additional file 1 Publications included in the bibliometric review based on appraisal of context country, study objectives and main conclusions

| Citation | Context country | Study objectives | Main conclusions |
| --- | --- | --- | --- |
| [Ali and Medhekar (86)](#_ENREF_86) | Thailand | Study the comparative experience of Bangladeshi patients seeking healthcare in Thailand | Better healthcare facilities, trained personnel and lower wait times have made Thailand an attractive option for Bangladeshi patients. |
| [Alsharif, Labonté et al. (75)](#_ENREF_75) | India | Study experiences of medical tourists in India, China, Jordan and the UAE | Medical tourism has worsened healthcare access for the poor in destination countries. |
| [Álvarez, Chanda et al. (66)](#_ENREF_66) | UK, India | Review stakeholder perspectives on the role of bilateral agreements in medical tourism between India and the UK | Political cost is an impediment to bilateral relationships for addressing concerns on medical tourism. |
| [Amodeo (47)](#_ENREF_47) | US | Review literature and international treaties relevant to medical tourism in the US and policy implications | There is lack of attention to the policy implications of medical tourism. |
| [Azmi and Awang (104)](#_ENREF_104) | Malaysia | Explore sustainability and stakeholder relationships in medical tourism | Full support and commitment from different stakeholders is needed for sustainable medical tourism. |
| [Béland and Zarzeczny (29)](#_ENREF_29) | US | Highlight the multifaceted relationship between medical tourism and national healthcare systems in the US and Canada | Characteristics of national health systems play a critical role in shaping demand for and impact of medical tourism. An institutionalist research agenda is proposed for policymakers to explore this nexus. |
| [Bergmark, Barr et al. (70)](#_ENREF_70) | Mexico | Explore the magnitude of and reasons for Mexican immigrants in US border states seeking health services in Mexico | Difficulty in accessing healthcare in the US and affinity for Mexican healthcare are key drivers for Mexican immigrants returning to Mexico for health services. |
| [Bloomer and O'Dowd (62)](#_ENREF_62) | UK | Explore causes of abortion tourism and legal barriers that restrict access to abortion services in Ireland | Moral conservatism has restricted access to abortion services in Ireland and led to rise of abortion tourism. |
| [Buchitchon (92)](#_ENREF_92) | Thailand | Study international and domestic moral standards contributing to the enactment of the Protection of Children Born from Assisted Reproductive Technology Act 2015 | Rational revision of the law is suggested to allow creation of human embryos for research purposes and lift restrictions on access to surrogacy arrangements, in consonance with both international and domestic moral concerns. |
| [Bustamante (48)](#_ENREF_48) | US | Identify barriers to transnational health services in the context of trade liberalization in North America | High regulation of healthcare services, resistance by organized interest groups, heterogeneity of healthcare regulations and lack of transnational reciprocity agreements are barriers to medical tourism. Better economic integration and healthcare reforms are necessary to overcome them. |
| [Chee (98)](#_ENREF_98) | Malaysia, Singapore | Review the comparative development and impact of medical tourism in Singapore and Malaysia | Medical tourism has led to commercialization of healthcare and furthered health inequities in both countries. Singapore has better managed reforms towards narrowing the public-private gap. |
| [Chin and Campbell (107)](#_ENREF_107) | Singapore | Critically appraise the practice of international transplant medicine in Singapore | Singapore has been pragmatic in addressing both its socioeconomic needs and the ethical concerns of transplant tourism. Suggestions to minimize the risk of abuse by medical tourists and organ traders are offered. |
| [Chomvilailuk and Srisomyong (85)](#_ENREF_85) | Thailand | Investigate the relationships between medical travelers’ perceptions of medical tourism and destination brand choice | Perceptions of medical travelers and their choice of destination brand are significantly correlated. |
| [Chuie-Hong and Sin-Ban (95)](#_ENREF_95) | Malaysia | Study the relationship between medical tourists, tourists' arrival and Malaysia My Second Home (MM2H) participants | Public-private cooperation is required to attract more medical tourists. Tourism development and health sector policies must be integrated. |
| [Cohen (116)](#_ENREF_116) | US | Examine risks and concerns on protecting US citizens traveling overseas for medical treatment | Outright prohibition or discouragement of medical tourism through insurance mandates may be problematic. Layered regulatory interventions are required to protect patients seeking medical care outside the US. |
| [Cohen (78)](#_ENREF_78) | India | Examine various regulatory approaches to medical tourism in home and destination countries | Regulation of medical tourism is complex, costly and challenging. There is need for more regulatory analysis to evaluate regulatory efficacy in terms of bioethical implications and enforcement feasibility in different governance contexts. |
| [Cortez (50)](#_ENREF_50) | US | Examine the potential of and impediments to cross-border health insurance plans in the US | Cross-border health insurance plans are a feasible and affordable alternative for underinsured US citizens and immigrants. |
| [Crooks, Whitmore et al. (37)](#_ENREF_37) | Canada | Study the health and safety challenges faced by informal caregiver companions of medical tourists | Practical advice provided by former caregiver companions can help in the design of informational interventions for prospective caregivers. |
| [De Jesus and Xiao (51)](#_ENREF_51) | US, Mexico | Study causes of cross-border health seeking by the US Hispanic population | Lack of medical insurance, lack of English proficiency and the perceived inefficiency of the American health system are key factors driving US Hispanic immigrants to seek healthcare in Mexico and other Latin American countries. |
| [Debata, Sree et al. (72)](#_ENREF_72) | India | Identify and classify key enablers of medical tourism and assess their effects | A range of enablers and their relationships are identified. A prescriptive framework is suggested to help policymakers identify the right enablers for promoting medical tourism. |
| [Einsiedel and Adamson (30)](#_ENREF_30) | Canada | Explore views on and willingness to avail stem cell tourism services, and the ethical and policy implications | Subjects are empathetic towards patients seeking stem cell therapy and willing to try it themselves when faced with critical illnesses in pursuit of hope for treatment, notwithstanding their awareness of its limitations, risks and ethical dilemmas. |
| [Fenton-Glynn (63)](#_ENREF_63) | UK | Review the efficacy of the English legislative regime in regulating international surrogacy | The English legislative regime is ineffective in regulating commercial payments related to international surrogacy. Better domestic regulation is required to locally cater to the demand for surrogacy arrangements, and prevent the exploitation of women overseas. |
| [Fulfer (35)](#_ENREF_35) | Canada | Explore the bioethical dimensions of Canadian policy on cross-border reproductive travel | Ban on commercialized reproduction, while well-intentioned, reinforces neocolonialism by neglecting the agency of surrogate mothers and egg donors in destination countries. |
| [Gan and Frederick (52)](#_ENREF_52) | US | Study motivations of American medical tourists to seek healthcare overseas | Inherent risks of medical travel, social factors and vacation are key factors in patient decisions to seek healthcare abroad. |
| [Ganguli and Ebrahim (106)](#_ENREF_106) | Singapore | Qualitatively identify and analyze Singapore's medical tourism competitiveness | Use of integrated medical tourism development strategies, sound government policies and proactive management practices have contributed to the competitiveness of Singapore as a medical tourism destination. |
| [Gill, Goldberg et al. (31)](#_ENREF_31) | Canada | Outline a policy to deter Canadian healthcare providers from engaging in transplant tourism and ensure optimal care for patients with end-stage organ failure | The policy provides a guiding framework for healthcare professionals in Canada involved in the care of patients who are likely to undergo or have undergone organ transplantation in a different country. |
| [Glazier, Danovitch et al. (59)](#_ENREF_59) | US | Review the policy for transplantation of deceased donor organs into nonresidents in the US | The policy ensures transparency, fixes responsibility and enhances public trust in transplant practices. |
| [Gola (77)](#_ENREF_77) | India | Identify the beneficiaries of policies promoting medical tourism and potential implications for the public health system | Lack of baseline data and adequate regulatory mechanisms for medical tourism in India have contributed to poor accountability, institutional fragmentation and non-coordination. |
| [Hazarika (76)](#_ENREF_76) | India | Outline the potential impact of medical tourism on India’s health workforce and health system | There is need for regulatory measures to address potential threats from medical tourism to the public health system. |
| [Hudson and Li (41)](#_ENREF_41) | US | Examine literature on domestic medical tourism in the US | There is lack of adequate research on consumer attitudes and beliefs regarding domestic medical tourism. |
| [Jarman and Truby (55)](#_ENREF_55) | US | Compares debates on medical tourism in the EU and US | The narrative is focused on cost in the US, whereas access and quality are key issues in EU debates. |
| [Johnson, Youngquist et al. (54)](#_ENREF_54) | US | Evaluate the potential of 24 country-level measures for predicting the quantum of medical travel to the US | The study provides a replicable, data-driven model for US hospitals to attract medical tourists. |
| [Johnston, Crooks et al. (38)](#_ENREF_38) | Canada | Understand Canadian patients’ involvement in medical tourism and its implications for public health | Factors influencing medical travel and the use of medical tourism facilitators were identified. A comprehensive policy response is necessary given the diverse motivations and mechanisms for medical travel. |
| [Johnston, Crooks et al. (69)](#_ENREF_69) | Mexico | Identify factors shaping the development of medical tourism in three Central American and Caribbean countries and their implications | Medical tourism is primarily driven by public investment agencies and the private health sector for economic benefit, with limited consideration of health equity concerns. |
| [Kirby (79)](#_ENREF_79) | India | Explore the exploitative nature of transnational surrogacy and measures to curb it | Transnational surrogacy is currently exploitative. A national regulatory framework that incorporates public education, enabled choice, protections for surrogate women and empowerment reforms is essential to address this issue. |
| [Klijs, Ormond et al. (99)](#_ENREF_99) | Malaysia | Evaluate the economic impact of medical tourism in Malaysia | Medical tourism has far greater economic impact on the non-medical sector than the medical sector. Indirect impacts are more substantial than direct ones. |
| [Lozanski (36)](#_ENREF_36) | Canada | Explore the impact of host country structures on transnational surrogacy, and compare Canadian domestic law with international policy | There is mismatch between Canadian domestic law and its policy on transnational commercial surrogacy which reinforces commodification and gender disparities. |
| [Manaf, Hussin et al. (96)](#_ENREF_96) | Malaysia | Evaluate medical tourism service quality and patient satisfaction | Medical staff quality is critical in shaping patient satisfaction from medical tourism and future intention to travel for treatment. |
| [Martin (56)](#_ENREF_56) | US | Analyze how ideologies of genetic determinism and consumer choices are embedded in the US fertility industry | Fertility industry practices cater to and reinforce consumer desire for genetic control. Globalization has contributed to the adoption and spread of selective genetic technologies transnationally, despite domestic regulations to limit their use in various countries. |
| [McGuinness and McHale (64)](#_ENREF_64) | UK | Explore legal responses to illicit organ tourism in England and Wales | The use of extra‐territorial jurisdiction to enforce provisions of the Human Tissue Act 2004, along with the Draft Council of Europe Convention against Trafficking in Human Organs, provide an effective legal response to control transnational commercial organ trade. |
| [Miyashita, Akaleephan et al. (91)](#_ENREF_91) | Thailand | Study the use of and need for health services amongst Japanese retirees in Thailand | The use of Thai medical services by long-stay Japanese retirees is mostly limited to acute medical conditions as they prefer to travel to Japan for long term medical care. |
| [Nemie and Kassim (101)](#_ENREF_101) | Malaysia | Explore opportunities and legal challenges of medical tourism in Malaysia | There is a need to assess the existing legal framework for regulating medical tourism in Malaysia to address its ethical and legal challenges. |
| [Ormond (97)](#_ENREF_97) | Malaysia | Contextualize the rise of medical tourism against ongoing healthcare privatization reform in Malaysia | Medical tourism in Malaysia has been politically leveraged to further a healthcare privatization reform agenda and a consumeristic national narrative. |
| [Ormond and Sulianti (94)](#_ENREF_94) | Malaysia | Examine South-South intra-regional medical travel through a study of motivations and practices of Indonesian medical travelers to Malaysia | Diverse socio-economic factors shape decisions of South-South intra-regional medical travelers. Informal social and economic networks play a critical role in supporting the formal medical travel industry. |
| [Oesterle, Johnson et al. (53)](#_ENREF_53) | US | Examine institutional environments in the US and EU that influence transnational medical tourism to develop a conceptual model of medical travel | Both patient-related and institutional factors play a role in patients’ decisions to seek medical tourism. The proposed framework can help in the design of more effective programs and governance arrangements. |
| [Palattiyil, Blyth et al. (80)](#_ENREF_80) | India | Review evidence on surrogacy in India and implications of the Assisted Reproductive Technology (Regulation) Bill | The legislation falls far short of recognized international ethical norms and principles on the governance of cross-border reproductive services and surrogacy. |
| [Pocock and Phua (87)](#_ENREF_87) | Thailand, Malaysia, Singapore | Provide a conceptual framework for assessing the policy implications of medical tourism for health systems based on cases of Thailand, Singapore and Malaysia | Medical tourism has exacerbated public-private inequities in healthcare, which regulatory and policy measures have failed to adequately address. The proposed framework can help policymakers better assess policy implications and design more effective regulatory interventions. |
| [Qadeer and Reddy (74)](#_ENREF_74) | India | Explore tertiary care physicians’ perceptions of medical tourism | The paper highlights key differences and similarities in perceptions of public and private sector physicians about medical tourism, and policy implications of the public-private disconnect. |
| [Rafighi, Poduval et al. (67)](#_ENREF_67) | UK | Explore NHS reform challenges and implications through perspectives of non-European Economic Area migrants and health advocates in London | Legislation restricting access to healthcare for immigrants can exacerbate existing health inequities among vulnerable populations in the UK. |
| [Rahman and Zailani (103)](#_ENREF_103) | Malaysia | Study the effectiveness and viability of Muslim-friendly medical tourism supply chain practices in Malaysia | Muslim-friendly supply-chain practices are found to improve overall organizational performance and can potentially help attract and cater to Muslim medical tourists. |
| [Renganathan, Vijayabanu et al. (71)](#_ENREF_71) | India | Study the relevance of blue ocean strategy in the development of medical tourism in India | A blue ocean strategy can be valuable in tapping into India’s healthcare market potential and developing medical tourism. |
| [Rhodes and Schiano (58)](#_ENREF_58) | US | Discuss ethical dilemmas of US transplant programs faced with patients receiving organ transplants from executed Chinese prisoners | Transplant policies must uphold the principle of beneficence and consider the needs and suffering of transplant patients without moral judgement. |
| [Robson (73)](#_ENREF_73) | India | Discuss positive and negative effects of medical tourism in India | Medical tourism has had mixed effects. There is need for policy intervention to facilitate the gains and mitigate adverse consequences. |
| [Runnels, Labonté et al. (39)](#_ENREF_39) | Canada | Understand Canadian physicians’ perspectives on and experiences of treating patients travelling overseas for medical treatment | Lack of information on treatment, complications arising from it and breach of care continuity are key concerns of physicians treating returning patients. |
| [Sandoval (81)](#_ENREF_81) | India | Discuss implications of the Assisted Reproductive Technology (Regulation) Bill for commercial surrogacy in India | There is lack of adequate protection for surrogate mothers and privileging of intending parents under the proposed legislation. |
| [Sankrusme (84)](#_ENREF_84) | Thailand | Determine effects of public management, service quality and overall attractiveness on foreigners’ perceptions of the Thai medical tourism industry | The quality of services and amenities provided are key factors in attracting medical tourists to Thailand. |
| [Sarojini, Marwah et al. (82)](#_ENREF_82) | India | Explore the nature, growth and challenges of the Assisted Reproductive Technologies industry in India | The prevalence of unethical practices calls for better regulation of the Assisted Reproductive Technologies industry. |
| [Sethna and Doull (32)](#_ENREF_32) | Canada | Trace abortion tourism in Canadian women | Travel to access abortion services across domestic and international boundaries is a long-established and widespread phenomenon that must be recognized as a form of medical tourism. |
| [Snyder, Crooks et al. (33)](#_ENREF_33) | Canada | Compare patient and academic perspectives on ethics of medical tourism | Though patient and academic perspectives overlap in many areas, there is scope for academic perspectives to be informed by patient perspectives for better resolution of the ethical challenges of medical tourism. |
| [Snyder, Johnston et al. (28)](#_ENREF_28) | Canada | Identify patterns of medical tourism in Canada and implications for preferential access to healthcare by Canadians | Medical tourism affects preferential access to healthcare by Canadians by bypassing domestic regulation, creating interjurisdictional friction in provisioning and undermining cohesion of the Canadian health system. |
| [Wahed (100)](#_ENREF_100) | Malaysia | Review legal and ethical issues of medical tourism in Malaysia | A uniform regulatory framework for medical tourism is required to address its legal and ethical challenges. |
| [Zarzeczny and Clark (34)](#_ENREF_34) | Canada | Explore regulator perspectives on and experiences of varied levels of physician involvement with stem cell tourism | There is need for clear policy guidance to physicians on how to respond to patient demands about unproven stem cell treatment, given their conflicting professional, legal and ethical obligations. |
